# Supplementary material for: Predictive Performance of the FRAX Tool Calibrated for Spain vs. an Age and Sex Model: Prospective Cohort Study with 9082 Women and Men Followed for up to 8 Years
Source: J Clin Med. 2022 Apr 25;11(9):2409. doi: 10.3390/jcm11092409 (PMC9101808; doi:10.3390/jcm11092409)
Supplement: Supplementary file 1 [file jcm-11-02409-s001.zip › jcm-1690062-supplementary.pdf]

Predictive Performance of the FRAX Tool Calibrated for Spain vs. an Age and Sex Model: Prospective Cohort Study with 9082 Women and Men Followed for up to 8 Years

Anibal García-Sempere, Isabel Hurtado, Salvador Peiró, Francisco Sánchez-Sáez, Yared Santaana, Clara Rodríguez-Bernal, Gabriel Sanfélix-Gimeno and Jose Sanfélix-Genovés

Supplementary Material S1. Age and Sex model: results of the 10-fold cross-validation and logistic equation.

10-fold cross-validation

Metric	Mean	n	Standard error
Accuracy	0.985	10	0.001
ROC_AUC	0.846	10	0.013

Logistic equation

$$logit(y) = -13.833 + 0.133 \cdot Age\; (years) + 0.661 \cdot Women$$

Supplementary Material S2. Calibration of observed versus predicted hip fracture by FRAX and the age and sex model by age and BMI strata.

Age strata

AGE and SEX

		n	Tasa	Prob esp	[95% Conf. Interval]		Obs	Esp	[95% Conf. Interval]		O/E	Ratio
**< 65**	5274	0.0030	0.0032	0.0021	0.0031	0.0032	16	17	16.48	17.08	16/17	0.95(0.94–0.97)
**65–69**	1329	0.0068	0.0104	0.0039	0.0102	0.0106	9	14	13.50	14.06	9/14	0.65(0.64–0.67)
**70–74**	941	0.0191	0.0202	0.0078	0.0197	0.0207	18	19	18.55	19.48	18/19	0.95(0.92–0.97)
**75–79**	820	0.0500	0.0384	0.0145	0.0374	0.0393	41	31	30.64	32.27	41/31	1.3(1.27–1.34)
**≥ 80**	718	0.0780	0.0821	0.0494	0.0785	0.0858	56	59	56.38	61.57	56/59	0.95(0.91–0.99)

FRAX

		n	Tasa	Prob esp	[95% Conf. Interval]		Obs	Esp	[95% Conf. Interval]		O/E	Ratio
**< 65**	5274	0.0030	0.0039	0.0042	0.0038	0.0040	16	21	20.00	21.19	16/21	0.78(0.76–0.8)
**65–69**	1329	0.0068	0.0118	0.0108	0.0112	0.0124	9	16	14.93	16.48	9/16	0.57(0.55–0.6)
**70–74**	941	0.0191	0.0248	0.0213	0.0235	0.0262	18	23	22.10	24.66	18/23	0.77(0.73–0.81)
**75–79**	820	0.0500	0.0467	0.0394	0.0440	0.0494	41	38	36.09	40.50	41/38	1.07(1.01–1.14)
**≥ 80**	718	0.0780	0.0585	0.0417	0.0555	0.0616	56	42	39.84	44.22	56/42	1.33(1.27–1.41)

BMI strata

AGE and SEX

		n	Tasa	Prob esp	[95% Conf. Interval]		Obs	Esp	[95% Conf. Interval]		O/E	Ratio
**< 25**	1695	0.0206	0.0157	0.0318	0.0142	0.0172	35	27	24.07	29.21	35/27	1.31(1.2–1.45)
**25–29**	3241	0.0164	0.0160	0.0277	0.0151	0.0170	53	52	48.92	55.11	53/52	1.02(0.96–1.08)
**≥ 29**	4146	0.0125	0.0148	0.0235	0.0141	0.0155	52	61	58.38	64.30	52/61	0.85(0.81–0.89)

FRAX

		n	Tasa	Prob esp	[95% Conf. Interval]		Obs	Esp	[95% Conf. Interval]		O/E	Ratio
**< 25**	1695	0.0206	0.0206	0.0345	0.0190	0.0222	35	35	32.14	37.70	35/35	1(0.93–1.09)
**25–29**	3241	0.0164	0.0164	0.0257	0.0155	0.0172	53	53	50.17	55.89	53/53	1(0.95–1.06)
**≥ 29**	4146	0.0125	0.0126	0.0214	0.0119	0.0132	52	52	49.35	54.75	52/52	1(0.95–1.05)
